# Nitric oxide modulates ATP-evoked currents in mouse Leydig cells

**DOI:** 10.1590/1414-431X20186693

**Published:** 2018-03-15

**Authors:** J.L. de Deus, A.L.A. Dagostin, W.A. Varanda

**Affiliations:** Departamento de Fisiologia, Faculdade de Medicina de Ribeirão Preto, Universidade de São Paulo, Ribeirão Preto, SP, Brasil

**Keywords:** Leydig cells, ATP, Purinergic receptor, Calcium, Nitric oxide, Whole cell patch clamp

## Abstract

Testosterone synthesis within Leydig cells is a calcium-dependent process. Intracellular calcium levels are regulated by different processes including ATP-activated P2X purinergic receptors, T-type Ca^2+^ channels modulated by the luteinizing hormone, and intracellular calcium storages recruited by a calcium-induced calcium release mechanism. On the other hand, nitric oxide (NO) is reported to have an inhibitory role in testosterone production. Based on these observations, we investigated the interaction between the purinergic and nitrergic systems in Leydig cells of adult mice. For this purpose, we recorded ATP-evoked currents in isolated Leydig cells using the whole cell patch clamp technique after treatment with L-NAME (300 μM and 1 mM), L-arginine (10, 100, 300, and 500 μM), ODQ (300 μM), and 8-Br-cGMP (100 μM). Our results show that NO produced by Leydig cells in basal conditions is insufficient to change the ATP-evoked currents and that extra NO provided by adding 300 μM L-arginine positively modulates the current through a mechanism involving the NO/cGMP signaling pathway. Thus, we report an interaction between the nitrergic and purinergic systems in Leydig cells and suggest that Ca^2+^ entry via the purinergic receptors can be regulated by NO.

## Introduction

The involvement of Ca^2+^ in Leydig cell's testosterone synthesis was suggested by Janszen et al. (1) by showing that the production of the hormone induced by the luteinizing hormone was dependent on the presence of Ca^2+^ in the extracellular solution. Other studies investigating the role of calcium ions in the steroidogenic process showed that application of ATP onto the cell increased the intracellular Ca^2+^ concentration (2) and the production of the steroid via activation of P2 receptors in Leydig cells (3). More recently our group demonstrated that P2X receptors, which have pharmacological and electrophysiological properties similar to P2X2 receptors, are present in the plasma membrane of mice Leydig cells (4). Moreover, Antonio et al. (5,6) showed that P2X2, P2X4, P2X6, P2X7 subunits are present in Leydig cells, possibly arranged as heterotrimers to form P2X2/4/6 receptors.

Therefore, the increase in intracellular calcium concentration ([Ca^2+^]_i_) due to P2X receptor activation is one of the processes that can enhance steroidogenesis in Leydig cells. On the other hand, nitric oxide (NO) has also been taken as a global modulator of this process. However, its role in testosterone production is not completely understood. Several studies have shown an inverse relationship between NO and testosterone production/secretion: inhibition of NO production induces an increase in the plasma levels of the hormone (7–11). In addition, Valenti et al. (12) showed a biphasic relationship in which higher concentrations of NO donors (S-nitroso-N-ncetyl-D, L-penicillamine, diethylamine NONOate, and diethylenetriamine NONOate) decreased testosterone production while lower concentrations of these compounds increased hormone levels. The formation of NO is catalyzed by three different enzymes, and two of them, the endothelial and the neuronal nitric oxide synthases (NOS) rely on an increased intracellular Ca^2+^ concentration to be activated (13) and are present in Leydig cells (14,15).

Different cell types have nitrergic and purinergic signaling pathways that interact with each other, determining the final physiological response. In the cochlear hair cells, ATP induces the production of NO by activation of P2 purinergic receptors (16). In rat hypothalamus, P2X2 receptors and neuronal NOS co-localize in the supraoptic nucleus and rostroventrolateral medulla neurons (17). In the thick ascending limb of the nephron, there is a decrease in NO production when either suramin or L-N-nitro-arginine methyl ester (L-NAME) are applied before application of extracellular ATP (18). In carotid body neurons, extracellular ATP increases intracellular Ca^2+^, triggering NO production (19). The above examples show that the purinergic and nitrergic systems act on several physiological processes, even though ATP and NO have different molecular identities.

Based on evidence showing the interaction between these systems in different cells, the aim of the present work was to study the relationship between the actions of NO and ATP-evoked purinergic currents in mice Leydig cells with the whole cell patch clamp technique.

## Material and Methods

### Cells

The protocols used in this study were conducted in accordance with the Ethical Principles on Animal Experimentation adopted by the National Council of Animal Experimentation Control (CONCEA) and approved by the Institutional Ethical Committee on Animal Experimentation of the Faculdade de Medicina de Ribeirão Preto, Universidade de São Paulo (#018/2013).

Leydig cells were obtained from 42-day-old male Swiss mice. The animals were killed by cervical dislocation and the testes were quickly removed and freed from fat and the surrounding tunica albuginea. They were placed in Hank's solution containing: 140 mM NaCl, 4.6 mM KCl, 1.6 mM CaCl_2_, 1.13 mM MgCl_2_, 10 mM Hepes, 10 D-glucose, and 5 mM NaHCO_3_, with osmolality ranging from 290–300 mOsm/kg and pH 7.4 adjusted with NaOH. Leydig cells were collected by mechanical dispersion (aspiration/suction with a 27-gauge needle and a syringe) of the testis with the same solution and plated on glass coverslips. For recordings, the coverslips were transferred to a perfusion chamber mounted on the stage of an inverted microscope (Axiovert 40 CFL, Carl Zeiss, Germany) no more than 4 h after isolation (4).

### Electrophysiological recordings

The patch clamp technique in the whole cell voltage clamp configuration was used for measuring the evoked purinergic currents. Micropipettes were pulled from borosilicate glass capillaries (Sutter Instruments Co., USA) in a P-97 puller (Sutter Instruments Co.) and had resistance ranging from 3–4 MΩ when backfilled with the internal solution containing 140 mM potassium gluconate, 10 mM KCl, 1 mM MgCl_2_, 10 mM Hepes, 1 mM EGTA, 0.75 mM CaCl_2_, 2 mM Na-ATP, and 0.25 mM Na-GTP, with osmolality between 285 and 295 mOsm/kg and pH 7.3 adjusted with KOH. Ionic currents were measured with an Axopatch 200B amplifier, filtered at 2 KHz and digitized at 5 KHz through a Digidata 1440A AD/DA converter (Molecular Devices, USA), controlled by the software PClamp 10. Cells with series resistance larger than 20 MΩ were discarded.

Drugs were diluted to the desired final concentration in the extracellular solution and applied to the cells via a rapid solution changer system (RSC - Bio-Logic Co., France). To construct the current versus voltage (I x V) plots, voltage ramps from −100 to +40 mV (500 ms long) were applied 800 ms after the beginning of the ATP superfusion. This delay is necessary to get the current response at its peak and was extended for another 100 ms after the ramp was terminated. Thus, the ATP application lasted for 1400 ms. This protocol was first applied with ATP only, 5 min after the whole cell configuration was achieved, and the subsequent ATP pulses were delivered in 5-min intervals. With L-arginine (10, 100, 300, and 500 µM), the protocol was repeated and the recordings were made 5 min after whole cell mode establishment and a final recording after a 15-min washout.

ATP-evoked currents triggered in cells treated with L-NAME (300 µM and 1 mM), 8-Br-GMPc (100 µM) and ODQ (300 µM) were recorded 5 min after the establishment of the whole cell configuration and after 10 min of treatment with each drug. ATP, L-arginine (L-NG monomethyl arginine), L-NAME, 8-Br-GMPc (8-bromoguanosine 3′,5′-cyclic monophosphate sodium) and ODQ (1H-(1,2,4) oxadiazolo[4,3-A]-quinoxalin-1-one) were purchased from Sigma-Aldrich Co. (USA).

### Statistical analysis

Data were analyzed by descriptive statistics and are reported as means±SE. The comparison between variables was performed using one-way ANOVA and Tukey’s post-test, and the unpaired *t*-test with a significance level of 5% (P≤0.05). Data analysis was done with Prism version 5.0 (GraphPad Software, USA) and Origin Pro 6.0 (Origin Lab Corporation, USA). For comparison between different treatments, current amplitudes were measured at −70 mV and averaged.

## Results

### Thorough washout and short ATP pulses prevented currents from strong desensitization

**Figure 1.**
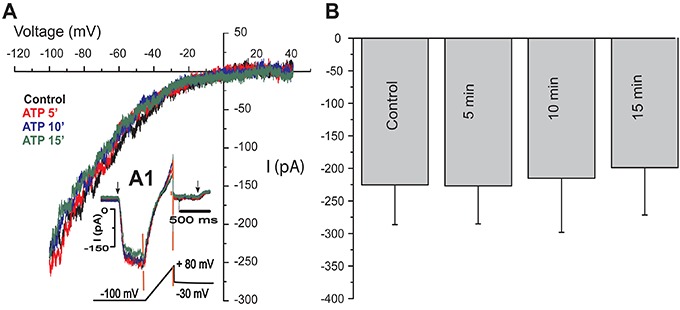
ATP-evoked currents did not show significant desensitization under the present experimental conditions. *A*, Current-voltage (I×V) plot obtained in response to 100 μM ATP application in control condition (first application - black) and after 5 (red), 10 (blue), and 15 min (green). The inset shows the voltage protocol used as well as the total current response. Note that application of ATP (arrows), with the cell held at −100 mV, induces a fast activating inward current. At the peak of the current response a voltage ramp going from −100 to +80 mV, with 500 ms duration, was applied and this corresponds to the IxV plot shown. The plot displays the response up to +40 mV only, because other currents start to activate beyond this point. This same procedure was used in all other experiments. *B*, Bar graph showing the means±SE of currents measured at −70 mV (n=7 cells). The cells were continuously superfused with control solution between ATP pulses. P≥0.05, ANOVA and Tukey post-test.

Purinergic receptors desensitize after long exposure to the agonist. Since our essays required repetitive cell exposure to ATP, we carried out an experiment to demonstrate that desensitization was not significant under our experimental conditions. Figure 1A shows superimposed IxV plots recorded during ATP applications separated by 5-min intervals. The responses are hardly distinguishable from one another. For comparison purposes, Figure 1B shows averaged values (n=7 cells) of the current amplitudes measured at −70 mV: control: −225.4±60.9, 5 min: −226.9±58.4, 10 min: −215.4±83.0, and 15 min: −198.9±72.7, pA; P=0.3. These results show that in our experimental conditions (ATP applied for 1400 ms + 5 min washout), the evoked purinergic currents suffered no significant desensitization. Therefore, any observed changes in amplitude can be associated with the pharmacological manipulation performed in each case.

### Basal NO production did not affect the ATP-evoked current

**Figure 2.**
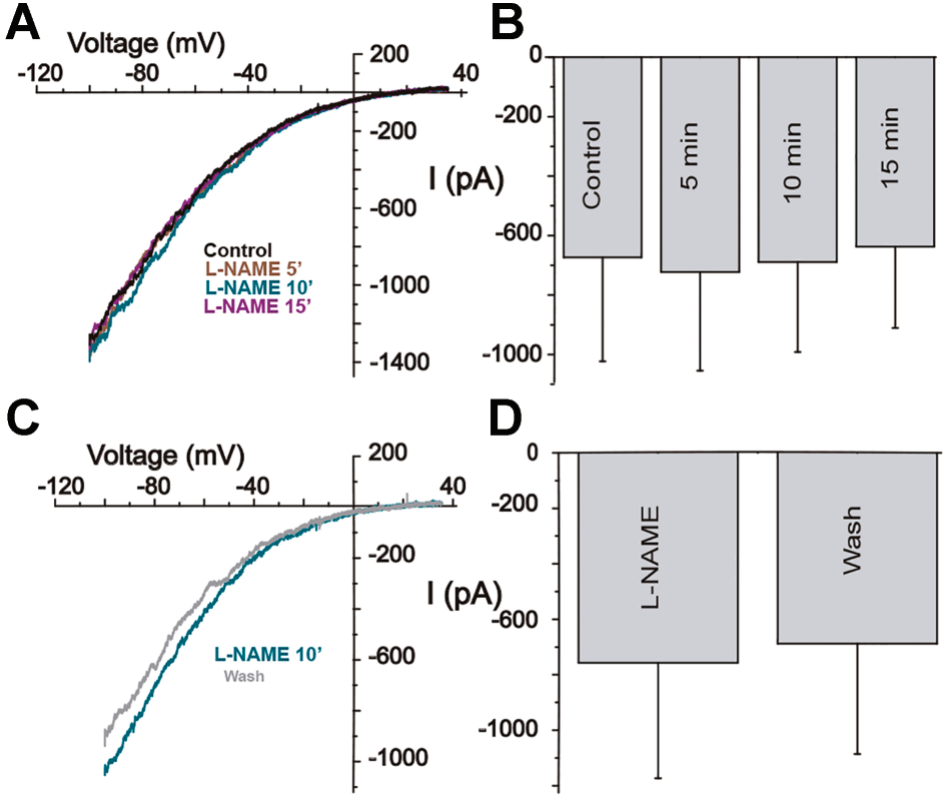
L-NAME did not impair purinergic currents. *A*, ATP-elicited currents I×V plot after treatment with 300 μM L-NAME for 5 (brown), 10 (light green) and 15 (purple) min. *C*, ATP-elicited currents IxV plot after treatment with 300 μM L-NAME for 10 min (light green) and after 15 min' wash (gray). *B* (n=5 cells) and *D* (n=5 cells), bar graphs showing the means±SE of the currents measured at −70 mV. No significant changes were observed in the amplitude of the currents. Panel B: P≥0.05, ANOVA and Tukey post-test; panel D: P≥0.05, unpaired *t*-test.

Since NOS isoforms are present in Leydig cells, we assumed that L-NAME, a non-selective NOS inhibitor, would block NO synthesis and, consequently, a possible basal nitrergic inhibition of purinergic currents. However, L-NAME did not impair the ATP-evoked currents after 5 (-722.5±331.5 pA), 10 (-688.9±302.8 pA), and 15 min (-637.0±273.5 pA) compared to control (-673.2±349.4 pA; P=0.95; n=5 cells (Figure 2A and B). Figure 2C and D reinforce this aspect, since the ATP-evoked currents measured after a 10 min incubation with 300 µM L-NAME are not significantly different from values acquired after washing out the drug for another 15 min (L-NAME: −756.7±416.8 pA; wash: −687.6±398.0 pA; P=0.4; n=5). Since we observed no significant difference, we may suggest that basal NO did not modulate the ATP-evoked current.

### Nitrergic modulation of ATP-evoked currents induced by L-arginine

**Figure 3.**
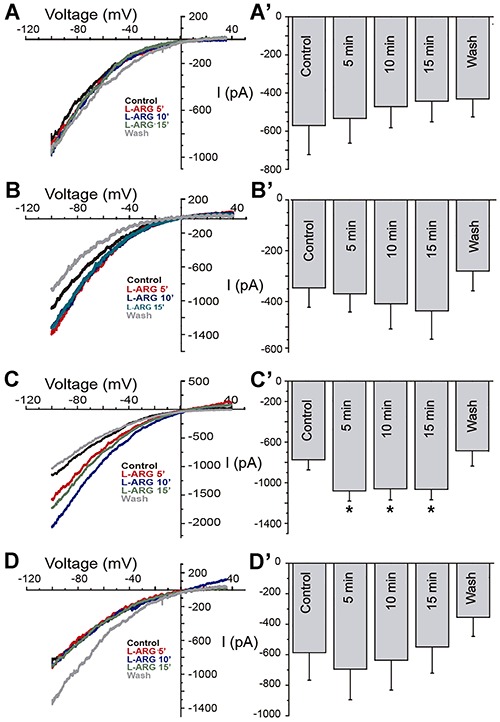
Effects of different concentrations of L-arginine on purinergic currents. *A*, *B*, *C* and *D*, Representative I×V plots for the ATP-elicited currents after the treatment with 10 μM (*A*), 100 μM (*B*), 300 μM (*C*), and 500 μM (*D*) L-arginine for 5 (red), 10 (blue), and 15 min (green) followed by 15 min (gray) washing. *A'* (n=9 cells), *B'* (n=8 cells), *C'* (n=7 cells), and *D'* (n=8 cells) show the means±SE of the current values at −70 mV for each group. Note the significant increase in the amplitude of the currents only with 300 μM L-arginine. *P≤0.05, n=7 cells (ANOVA and Tukey post-test).

Since basal NO production did not affect the ATP-evoked current amplitudes, we gradually increased the bath concentration of L-arginine to increase NO production and observe its effect on the purinergic currents (Figure 3A-D with 10, 100, 300, and 500 µM L-arginine). As can be seen, of the different concentrations of L-arginine used, only 300 µM significantly altered the purinergic currents (Figure 3C'). The ATP-evoked current amplitudes increased after treatment with L-arginine in 5, 10, and 15 min, and this effect was reversed upon L-arginine washout (P≤0.05)

### ATP-evoked currents were modulated by NO

**Figure 4.**
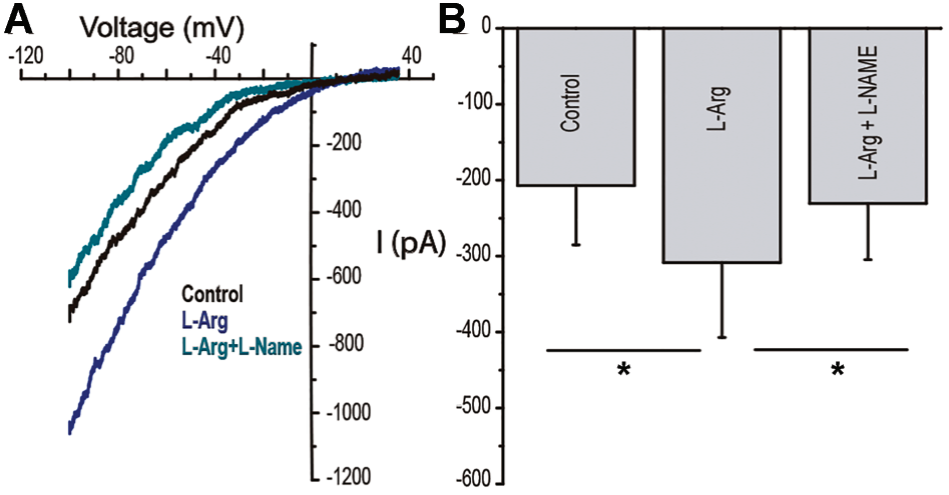
Three hundred μM L-arginine modulated purinergic currents. *A*, I×V relationship of ATP-evoked currents in control (black), after 10 min L-arginine incubation (blue), and after 10 min treatment with 300 µM L-arginine + 1 mM L-NAME (light green). The bar graph in *B* shows the mean±SE amplitude of the ATP current at −70 mV. *P≤0.05, n=7 cells (ANOVA and Tukey post-test).

This set of experiments was performed to confirm whether the increase in the ATP-evoked current induced by 300 µM L-arginine was due to an action of L-arginine itself or to an increased NOS activity and consequently to NO. Figure 4 shows that 10 min incubation with 300 µM L-arginine induced a significant increase in the ATP-evoked current. Nevertheless, a significant decrease was observed after 10 min superfusion of the cell with 300 µM L-arginine associated with 1 mM L-NAME (control: −206.9±78.2; L-arginine: −308.3±98.6; L-arginine + L-NAME: −230.7± 74.1 pA;. P≤0.05, n=7), confirming that NO was directly responsible for modulating the purinergic currents

### NO modulated the ATP-induced current through a cGMP pathway

**Figure 5.**
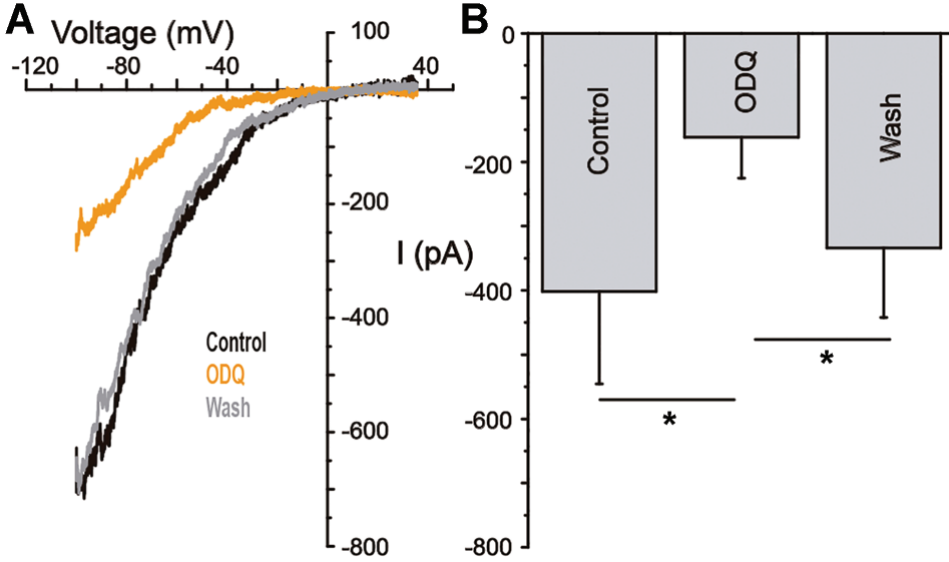
cGMP modulated purinergic currents. *A*, I×V relationship for ATP-evoked currents in control condition (black), after 10 min ODQ incubation (orange), and after washing with Hank's solution (gray). The bar graph in *B* shows the mean±SE amplitude for the currents measured at −70 mV. Note the significant amplitude decrease after ODQ incubation and its recovery after washing with Hank's solution. *P≤0.05, n=5 cells (ANOVA and Tukey post-test).

It is widely known that NO can regulate Ca^2+^ homeostasis through a NO-cGMP-PKG pathway playing different roles in different tissues. To verify if the cGMP modulates purinergic currents in Leydig cells, we repeated the ATP stimulation protocol in the presence of 300 μM ODQ, a selective guanylate cyclase inhibitor. Figure 5 shows ATP-evoked currents recorded before ODQ, after 10 min of incubation with ODQ and after washing out ODQ (Figure 5A) and the average current amplitudes measured at −70 mV (Figure 5B). ODQ caused a significant decrease in the ATP-evoked current amplitudes at −70 mV, which were readily reversed upon ODQ washout (control: −401.8±143.5 pA; 10 min ODQ incubation: −161.6±63.6 pA; washout: −333.9±108.3 pA; P≤0.05, n=6).

**Figure 6.**
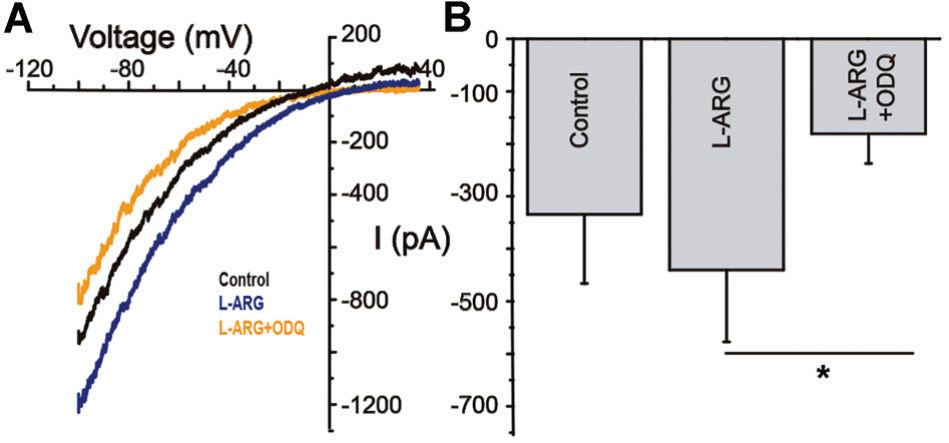
Purinergic modulation is dependent of the soluble guanylate cyclase (sGC). *A*, I×V plot of the ATP-evoked currents in control condition (black), after 10 min of incubation with L-arginine (blue), and after 10 min of treatment with L-arginine + ODQ (orange). The bar graph in *B* shows the means±SE amplitude for the currents measured at −70 mV. We observed a significant decrease in current amplitudes after sGC inhibition. *P≤0.05, n=6 cells (ANOVA and Tukey post-test).

To investigate whether the nitrergic modulation of purinergic current is dependent on the activation of the guanylate cyclase (GC) enzyme, we recorded ATP-induced currents in control conditions, 10 min after treatment with L-arginine, and 10 min after superfusion with L-arginine associated with ODQ (Figure 6A). As seen before, there was an increase in the ATP current upon treatment with L-arginine compared to control, and a clear decrease in the amplitude upon ODQ application (Figure 6B; control: −334.7±131.3; L-arginine: −440.6±136.7; L-arginine+ODQ: −181±58.4 pA; P≤0.05).

**Figure 7.**
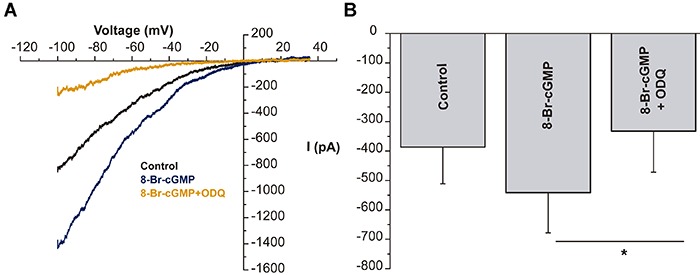
NO modulates purinergic current by the cGMP pathway. *A*, I×V plots for the ATP-evoked currents in control condition (black), after 10 min of treatment with 8-Br-cGMP (dark blue), and after 10 min incubation with 8-Br-cGMP + ODQ (orange). The bar graph in *B* shows the mean±SE amplitude of the ATP current at −70 mV. Note the significant amplitude decrease after the soluble guanylate cyclase (sGC) enzyme inhibition. *P≤0.05, n=6 cells (ANOVA and Tukey post-test).

To confirm the involvement of cGMP in the modulation of the purinergic currents, we repeated the measurements after 10 min of treatment with 100 μM 8-Br-cGMP, a membrane permeable cGMP analog, and after 10 min of treatment with 8-Br-cGMP associated with 300 μM ODQ (Figure 7A). Figure 7 shows that 8-Br-cGMP enhanced the ATP currents *per se* (control: −386.5±124.8 pA; 8-Br-cGMP: −541.5 ±137.1 pA). As expected, GC blockade by ODQ led to a significant decrease in the current amplitude even when the cells were treated with 8-Br-cGMP (-332.4± 139.4 pA; P≤0.05; Figure 7B).

## Discussion

In this study, we demonstrated that purinergic and nitrergic systems interact in mice Leydig cells. Our results show that the purinergic current amplitudes were not directly modulated by NO produced in basal conditions but that activation of NOS with L-arginine modified the ATP-induced current. Interestingly, L-arginine had its strongest effect on the purinergic currents at a concentration of 300 µM, since significant changes in the amplitude of currents at lower (10 e 100 µM) as well as higher concentrations (500 µM) were not observed. The effect is mediated by GC, since ODQ blocks the effects of both L-arginine and of 8-bromo-cGMP. Even though the rate of desensitization of purinergic currents in Leydig cells is dependent on the concentration and exposure time to ATP (4), we overcame this issue with a specifically designed ATP application protocol. First ATP was applied for a short time (1400 ms) avoiding a major desensitization component. Second, we chose to apply a voltage ramp at the peak of the ATP effect, avoiding voltage pulses with long durations. Figure 1A clearly shows that desensitization was not of significance under these conditions.

Treating the cells with L-NAME, a NOS inhibitor, revealed that basal NO, at least in Leydig cells, was not enough to significantly modify the amplitude of the ATP-induced currents. This finding agrees with other results showing no significant effects of L-arginine when used in the lower range of concentrations (Figure 3A). This sort of effect is also seen in endothelial cells from blood vessels, where the plasma concentration of L-arginine (100 to 800 µM) is more than enough to support NO synthesis (nNOS K_m_: 1.4-2.2 µM, inducible and endothelial NOS: 2.8-32.3 and 2.9 µM, respectively) (20). Nevertheless, further increases in the extracellular concentration of L-arginine raises NO synthesis even more. This phenomenon is described as the "arginine paradox": extra L-arginine is necessary for NO synthesis even in the presence of intracellular saturating levels of the substrate (21).

Concerning the additional NO demand to induce modulation of the ATP-evoked current, we observed that 300 µM L-arginine was able to change the currents during our observation time (i.e., 15 min). Also, we can assure that the effect was due to the presence of the substrate, since the washing out of L-arginine brought the currents back to its initial values. A different response was observed for testosterone production, since low concentrations of NO donors (SNAP, DEA/NO, and DETA/NO) increase and high concentrations decrease hormone production (12).

The substrate modulatory effect on the ATP currents is reinforced by the significant reduction of their amplitude when the cells were incubated in the concomitant presence of L-NAME and L-arginine. In this aspect, studies related to the effects of NO on hormone production by Leydig cells are contradictory: Gaytán et al. (22) showed a reduction in testosterone production after a 1 g/kg L-AME (exogenous substrate of NOS) intraperitoneal injection; Weissman et al. (23), on the other hand, described no alteration of testosterone production in either the absence or presence of the substrate. We may argue that these antagonistic observations may be due to differences in experimental protocols (substrate concentration, cell condition, methodologies used).

Treatment of Leydig cells with ODQ reduced the amplitude of the ATP-evoked currents. After washout of the GC inhibitor, the currents returned to their original values. Also, treatment of the cells with 8Br-cGMP confirmed the direct involvement of cGMP in modulating the ATP-induced currents (Figure 7). The effects of soluble guanylate cyclase (sGC) and cGMP were studied in human Leydig cells by Davidoff et al. (24). More recently, Andric et al. (25) identified, *in vitro*, the α1 and β1 sGC subunits in Leydig cells. They also showed that these cells produce less testosterone and have smaller concentration of cGMP when incubated with the sGC inhibitor NS2028 associated to the NO donor DPTA. Our data, showing a modulatory role of sGC and cGMP on the ATP-evoked currents in mice Leydig cells, are in accordance with the above results.

Our findings are also in accordance with a previous study by Shen et al. (26), demonstrating that Ca^2+^ homeostasis may be regulated by NO through a NO-cGMP-PKG pathway. After treatment of the cells with L-arginine and ODQ, the currents were significantly diminished, unveiling the contributions of sGC and NO/cGMP signaling pathway to the modulatory effect exerted by NO. Del Punta et al. (10) showed that there is no increase in the cGMP production with or without GC after the incubation of Leydig cells with the NO donor DEA/NO. Matsunobu and Schacht (27) showed a decrease in intracellular [Ca^2+^] when cochlear cells were treated with DEA/NO, SNP, and 8Br-cGMP, and an increase when GC was blocked by LY83583. However, Khurana et al. (28) showed that atrial natriuretic factor (ANF), cerebral natriuretic factor (CNF) and type C natriuretic peptide (CNP) augmented the GC activity and cGMP in Leydig cells. They also observed that the cGMP inhibitor LY83583 diminished testosterone production. Taken together, our data and that of Khurana et al. (28) clearly show an involvement of sGC and GC, respectively, and the participation of cGMP in the process, reinforcing the role played by this second messenger in steroidogenesis directly and indirectly through the modulation of purinergic currents.
